# Diabetes care among urban women in Soweto, South Africa: a qualitative study

**DOI:** 10.1186/s12889-015-2615-3

**Published:** 2015-12-26

**Authors:** Emily Mendenhall, Shane A. Norris

**Affiliations:** Walsh School of Foreign Service, Georgetown University, Intercultural Center Room 513, 37th and O St., NW, Washington, DC USA; MRC/Wits Developmental Pathways for Health Research Unit, University of the Witwatersrand, Johannesburg, South Africa

**Keywords:** Diabetes, Diabetes management, Health systems, Diet, Diabetes education, South Africa, Women

## Abstract

**Background:**

Escalation of non-communicable diseases such as Type 2 diabetes among low-income populations in low- and middle-income countries presents challenges for health systems. Yet, very little is known about low-income people’s diabetes care experiences in such contexts. One of the greatest challenges of diabetes care in such contexts is providing care for those who face poverty, poor healthcare access, and concurrent physical and mental conditions. This article investigates women’s experiences with diabetes care in Soweto, a township of Johannesburg, South Africa.

**Methods:**

This study involved caregivers for children enrolled in the Birth to Twenty (Bt20) cohort study initiated in 1990. Enrolled in the study for more than two decades, women previously diagnosed with type 2 diabetes were invited to participate. We conducted 27 in depth interviews around issues of stress, diabetes, mental health, and diabetes care. We transcribed interviews and used content analysis to analyze emergent themes into three categories: counseling, treatment, and social support.

**Results:**

First, counseling focused on nutrition but very little on exercise, and women had limited understanding of what was diabetes or what they should do to control it. Second, women were inconsistent with reporting their diabetes treatment routines, both with adhering to medicines and seeking treatments. They identified structural barriers as overcrowded clinics and poor access to medicines as impeding adherence to treatment. Finally, women identified support from their families and friends and recognized stress associated with these relationships around food (e.g., we’re not eating that!) and diabetes stigma.

**Conclusions:**

Effective diabetes education and management in the clinical setting will require systematic changes to healthcare. Inconsistencies across public and private health systems with regards to diabetes counseling, drug availability, quality of care, and patient wait times indicate patients will forego a clinical visit in lieu of diabetes self-care. For example, structural barriers in the public health system undermine medication adherence. With a stronger national emphasis in healthcare on diabetes counseling and management such systemic issues should be reshaped to ensure patients have access to essential medication and services.

## Background

Thirty-eight million people die annually from non-communicable diseases (NCDs), such as type 2 diabetes, heart disease, and hypertension, and three-quarters of these global deaths occur in low- and middle-income countries (LMICs) [1]. Although historically NCDs have afflicted affluent population, low-income populations increasingly present higher incidence, prevalence, and mortality as a result of NCDs [2, 3]. Escalation of NCDs in LMICs, and in particular among the poor in such contexts, pose not only extraordinary economic risk [4] but also unprecedented challenges to health systems already overburdened by infectious diseases, including HIV/AIDS [5].

South Africa is a country undergoing rapid health system transformation [5] that historically presented challenges for NCD care because of its great systemic inequalities [6] as well as its extraordinary burden of HIV/AIDS and tuberculosis [7, 8]. Yet, South Africa holds the second largest number of people living with type 2 diabetes in sub-Saharan Africa [3], with 2.7 million people, or 5 % of the population affected by diabetes [9]. The largest concentration of diabetes cases in South Africa are among low-income groups [10], with recent studies estimating 13.1 % diabetes prevalence among urban Blacks in the Cape [11] and 14.1 % diabetes prevalence among urban Black women in Soweto [12], low-income neighborhoods in Cape Town and Johannesburg, respectively. Increases in diabetes among the poor facilitate increased clustering of diabetes with other conditions [13], including mental illnesses [10], such as depression [14], and infectious diseases [8], such as HIV and tuberculosis [7]. Co-occurring conditions pose challenges for how people experience both diabetes and diabetes care [15, 16].

This article investigates low-income Black women’s experiences with diabetes care in Soweto, South Africa. Elsewhere we have argued that low-income Black women with diabetes hold low levels of knowledge about what is diabetes, its causes, and how one treats the disease [15]. This may be a reflection of their poor access to healthcare [10], mistrust of the healthcare system [17], or impediments to effective diabetes self-care and behavior change, such as poor health literacy, a lack of self-efficacy, and perceived social support [18]. It may also be a reflection of the public healthcare delivery system, whereby 64 % of South Africans depend upon the public sector for all healthcare needs [6]. Escalation of diabetes among low-income populations, therefore, places extraordinary demand for education and diabetes care delivery upon government hospitals and clinics [10]. Effective low-cost solutions for NCD care are sorely needed that move from the clinic to the community [19].

Systematic inequalities in health care delivery have muted South Africa’s response to NCD care nationally [10] and contributed to low-utilization of healthcare for NCDs [17, 20]. There are some exemplar efforts, however, to carry out effective NCD care. For example, community based interventions for diabetes control and management, like the Kgatelopele program in Gauteng Province or the Community Health Intervention Program, deliver medication and routine services to patients’ homes [10]. Others have demonstrated that mobile phone technologies, such as SMS-messaging [21], improve NCD care. Although community health worker programs seem to be effective at improving control of diabetes, systemic factors like too few doctor visits and insufficient patient monitoring by clinic staff hinder programs’ activities [22]. Moreover, doctors express lifestyle changes as the major obstacles to diabetes care [23], placing blame on the patients as opposed to the system. This contrasts to qualitative studies with women living with diabetes who mistrust the healthcare system and find it unreliable [17].

This article qualitatively examines perceptions about diabetes care of women with diabetes enrolled in the Birth to Twenty Plus (Bt20) Cohort in Soweto. Most women sought care at a public hospital or clinic near their home. Some received public care as well as private, and others received only private healthcare (such as through their employer). For this analysis, we examine three major themes related to diabetes care, including counseling, treatment, and social support, in order to understand challenges and opportunities for enhancing diabetes care for low-income women seeking care in South Africa’s public health system. Our qualitative insight provides a window through which South African policy-makers and practitioners working with low-income populations with diabetes can envision improved diabetes care.

## Methods

### Participants

Women were recruited from the Birth to Twenty Plus (Bt20) cohort study at Chris Hani Baragwanath Hospital (“Bara”) in Soweto, South Africa. The history and composition of Bt20, previously known as “Mandela’s Children” and “Birth to Ten”, is explained in depth elsewhere [24]. Notably in this paper we use the term ‘Black’ to describe the women interviewed for this study; we do so knowing that ‘Black’ is a political category instated during apartheid and that important cultural nuances linked with ethnicity exist in South Africa [25]. The Black women who participated in this study represent various regions of South Africa and are unified by their resettlement in Soweto, long-term residence there, and enrolment in the Bt20 cohort study.

Seventy-three adult Bt20 women of more than 1,000 women interviewed in 2010 self-reported previous diagnosis of Type 2 diabetes. These 73 women were contacted to participate in this study by phone; women did not participate if they were unavailable during the interview period, unreachable by phone, unable to travel, or deceased. We excluded active substance abusers and individuals who had severely disabling diabetes complications, cognitive impairment, or psychosis severe enough to interfere with participation in the interview.

We conducted 27 face-to-face interviews at Bara between November and December of 2012. Nineteen interviews were conducted in English by the first author and a multi-lingual research assistant (RA) conducted eight interviews in Zulu or Sesotho. The first author observed every non-English interview to ensure consistency across the interviews and discussed each of these interviews in depth with the RA. The RA was the first contact for each study participant by inviting them to participate and scheduling an interview time. After providing informed consent, women participated in a 60-90 min in-depth qualitative interview followed by surveys of demographics, mental health, and physical disease [15]. All interviews were audio-recorded and women were compensated 50 ZAR (around 5.88 USD) for transportation costs. All data collection received clearance by the University of the Witwatersrand Human Ethics Committee (Clearance number M121059). Data collection, analysis, and reporting adheres to the RATS guidelines.

### Data collection

The qualitative data presented in this article come from a larger interview around stress and diabetes. Each narrative interview began with: “Can you describe a typical day?” The interview then shifted to address stress, social relationships, family, and community problems or support systems. We concluded the interview with questions around healthcare experiences, such as “Can you tell me how you care for your diabetes?”, “What is a typical doctor’s visit like? Can you walk me through it?”, “Where do you get information about how to manage your diabetes?”, “How frequently do you receive diabetes care?”, and “Do you ever seek diabetes care from traditional healers?” These questions centered on personal experiences with diabetes care and perceptions of social and systemic barriers to care.

### Data analysis

A research assistant transcribed the qualitative interviews and translated those conducted in Sesotho and Zulu into English. We used an iterative analytical approach. First, we identified emergent themes, developed a codebook, and coded the 27 transcripts for common themes. Through critical analysis of emergent themes and patterns around heath-seeking and diabetes care-experiences, we recognized that three orienting factors emerged that women prioritized in diabetes care: counseling, treatment, and social support. We extracted representative quotes that communicate the challenges and opportunities associated with each theme. We present the results according to these three overarching themes to illustrate women’s views on challenges and opportunities for diabetes care in Soweto.

## Results

The 27 women in this study were on average 59 years (43-79 years) of age and had diabetes for ten years (Table 1). Most women completed less than 12 years of schooling (63 %) and around half relied on a monthly government pension or less. Most had no health insurance (67 %) and sought healthcare from government health facilities (78 %). All but two women reported one or more co-morbidity with diabetes, such as hypertension, arthritis, and depression.

**Table 1:** Sample characteristics

	Total (*n* = 27)
Age (mean, ±SD)	59 ± 9.3 years
Duration of Diabetes (mean, ±SD)	10 ± 6.0 years
Education (*n*, %)	
Less than Matric (12 years)	17 (63 %)
Completed Matric and/or More	10 (37 %)
Income (*n*, %)	
Pension or Less (R1,260)	13 (48 %)
More than Pension	14 (52 %)
Health Insurance (*n*, %)	
None	18 (67 %)
Health Insurance	9 (33 %)
Health Center (*n*, %)	
Government	21 (78 %)
Private Provider	6 (22 %)

### Counseling and diabetes education: sources and perspectives

With two-thirds of the women seeking care at public clinics, it was not surprising that the majority of women described receiving diabetes information “at the clinic” (meaning a community clinic) or “at Bara” (meaning the public tertiary hospital), or an associated support group. Women often identified doctors as educators: “The doctor normally tells me what to do and he usually gives me newsletters and magazines on diabetes”. Some women engaged with their doctor and others at the clinic:

The doctor gave me nutritional information and my colleagues at the clinic gave me further information and a lot of support with that. I also read a lot and was a member of “Diabetics South Africa” and I received all those magazines and information booklets. […] My doctor also gave me some pamphlets on nutritional information the last time I visited him. I read a lot and I also listen to the media. There is a […] lot of information on Dr. Oz’s show. He is very helpful and I watch his show every day.

Common sources of information about diabetes were “the newspaper”, “television”, and “Dr. Oz” (a popular US talk show). An elderly woman described: “There is a lot of information on TV about diabetes […] if there is still more information that I need, I usually consult the nurses at the clinic”. This exemplifies how women receive and prioritize diabetes counseling from multiple sources.

Nevertheless, women also stated that health providers “didn’t give me nutrition information” and therefore sought diabetes information from family, friends, and other patients. One woman described: “They [doctors] never told me much […] I have only heard from the other patients about what to eat and what to avoid”. Another elderly woman explained:

I saw what my mother and brother were doing for their diabetes [and] I told myself I will follow their routines for diabetes. At the clinic we were told to group ourselves, buy the same t-shirts; diabetes ones, orange in color and group ourselves to exercise as a group. Always the doctors are telling us the same thing to do when exercising, so that we keep on doing the right thing.

Women diagnosed with diabetes at Bara reported that meeting with a dietician before they left the hospital was a fundamental source of diabetes education, as exemplified by the following quote:

They told me mainly about the kind of food I have to eat, the butter to use. They also told me that I won’t find that butter in the same fridge as margarine, in store, but where they pack yogurts. Also that I must start eating more vegetables, that I should do away with fatty meat or food. Should only eat 4 grapes at a time, only half the banana, half orange and so on. […] But I’m battling to leave the pap. I know it has a lot of starch.

Despite education soon after diagnosis, few women continued to seek diabetes information. Many women “care for my diabetes on my own” and others “just keep to what the doctor tells me”. Many women reported only seeking care when they “feel bad,” exemplified by the following woman’s description of self-care: “At the moment I feel that sugar diabetes is no longer a concern to me. I have come to terms with my situation and I understand that I just need to be faithful to my treatment [meaning medication]”. As such, many women prioritized self-care over medical care, even during a “diabetes crisis”.

### Treatment: food, exercise, care-seeking, and medication

Eating the right foods was described both as a necessity and struggle. Some identified good eating habits as caring for diabetes, and others explicitly stated that eating healthy was not easy in Soweto. An elderly woman said: “Junk food is a common treat in the Township. Bunny chows with Vienna’s, cheese, bologna and chips are the order of the day mostly during lunchtime. I have had to forego all of those including those little chip snacks sold in plastics by most vendors”. Another expounded on how she places cultural value in high-caloric foods like meat and “pap”, the staple carbohydrate:

I like meat with all my heart, I must be honest. So, it’s even sometimes difficult for me to remove the fat; it becomes so tasteful I must be honest, it’s very difficult for me to leave fat [… and] to eat plain food without fat. Again, I had a problem leaving our stable food “pap” as prescribed by the doctor.

Others struggled to buy healthy foods recommended by their physicians because of cost, demonstrating diabetes food insecurity [15]. One woman stated, “I do not have the money to buy the necessary food [for my diabetes]”. Others emphasized links between stress, eating, and glucose control: “when I am stressed my glucose spikes” and “When you get stress, your sugar is uncontrollable”.

Exercise is a common aspect of diabetes treatment, and most women exercised exclusively by doing housework or walking intervals of the journey to work. This was described as “I don’t exercise much but I make up for it with household chores and a bit of gardening”; “I exercise because when I go to work I take one taxi and when I get off I walk the remainder of the trip until I get to work”; and “I care for the baby so I am always moving around the house”. These quotes illustrate that exercise apart from daily routine, such as walking for exercise, was an uncommon cultural practice.

Navigating public, private, and traditional healing systems for diabetes care was a central theme. Although one in three women had health insurance, many chose the public option over the private one. One woman said, “I’d even advise my friends with medical aids that they are wasting it, because here at Bara they’d check you and refer you to the doctor, like the ones at St John’s every six months”. She went on to explain her mistrust of physicians at private hospitals: “I wouldn’t mention the doctors at the private hospitals because they accumulate money; they will treat all your diseases as long as you pay them and pop money into their pharmacies”. Nevertheless, there were many systemic barriers through the public hospital system, from long waiting periods to meet with the doctor to poor hospital facilities. One woman said: “The clinic is ill-equipped because at times there aren’t enough gadgets to test either our sugar levels or just our blood pressure. At times the toilets are out of order”. Wait time was the most common complaint: “Seeing the doctor requires some patience and I have become used to the long queues. I get there at about 7 AM only to see the doctor at 12 noon”.

Fewer women chose non-public care options, including private healthcare and traditional healers. One in three participants accessed private healthcare but only a small number preferred private healthcare. One woman stated: “I think the [private] doctor is better. Yes, the clinic is free of charge but the treatment is abysmal. They shout at us. It takes forever before you are attended to. The queue is very long there”. Three women mentioned that they sought care from a traditional healer for their diabetes. An elderly woman who sought care from a faith healer explained, “I have been to a faith healer and asked for special diabetes prayers. It was during the time that I was seriously sick and I believe that he helped me as I feel a lot better now”. Another woman described an experience seeking care from a traditional healer negatively: “They blamed it on spirits [and] the traditional healer made it worse. It was only when I went to the doctor that I was diagnosed with hypertension and I only felt better when I was given treatment for it”. Most stated they did not seek care from traditional healers “because I trust God and not them”. Many separated diabetes care from what the traditional healers were capable of treating, such as the following woman who said: “I don’t believe in them because […] they’d never diagnose you with treatment for sugar diabetes and the like. The only thing is to tell you about witchcraft”.

Medication was another systemic divide between public and private diabetes care. Many referenced medicine as a key aspect of private insurance that was preferred, largely because “I now have my medication delivered at home so I hardly go to the doctor unless I need a checkup”. Many seeking care at the public clinic indicated: “I first went to the community clinic, but there’s usually no medication available, so they tell you to buy it”. Delivering medication to the home was important because it alleviated stress, and routinely taking one’s diabetes medication was often cited as stressful. One woman exemplified this in her statement, “Forgetting to take my tablets could be the only stressful thing in this regard and it does happen when I am very busy”.

### Social support: family and friends

Social support is essential for diabetes care. Women described positive social support to be talking about diabetes, walking together, and discussing problems with friends and family. One woman explained, “People have been supportive [of my diabetes]. My neighbor now also has it and we are always talking about it together and I enjoy being around her. We usually share a laugh about it sometimes”. Yet, modeling one’s personal diabetes journey often was related to a family member’s experience. A younger woman stated, “I am always stressed even more so because my mom has been a diabetic since 1989 and I can’t bear the thought of going through the same pain as her. She suffers from many other ailments other than diabetes, arthritis, weight loss, every night she complains about pain in her legs amongst other things”. An older woman grieved, “Some feel that it’s a death panel. They are still whispering about someone having the diabetes, saying that your legs are going to be cut and all sorts of things. I think that one of the elements contributing to us feeling scared of the diseases and try to hide it”. Indeed, many sought social support to quell fear or stress emanating from diabetes itself. As such, diabetes is a very social experience; when asking one woman if diabetes changed her life in any way, she responded: “My diet has changed and my overall body condition also changed. My relationship with my husband also changed and I even had to retire from work due to diabetes”.

Others suggested that diabetes stress was largely associated with behavior change. One woman said, “I do [have stress due to diabetes] but only as far as wishing that I wasn’t a diabetic. It limits me to some extent and at times I envy those that can still eat ice cream” and she went on to explain, “I must also keep my temper in check as I can’t afford to lose it otherwise I risk escalating my blood sugar level and blood pressure”. Social support also became realized in eating foods that were compatible with health providers’ recommendations. Family expectations that mothers eat with (as opposed to special foods apart from) the family were a common stress. One woman described this stress: “I think the food part is the most stressful one. I am the only one that’s diabetic at my home and the kids prepare food in a manner that is not always compatible with my condition. Spicy food upsets my health”.

## Discussion

This paper examines many opportunities and challenges for diabetes care for low-income populations in South Africa. First, we found that nutrition education immediately after diagnosis was memorable and made an impact on how many women care for diabetes; however, women received competing sources of information from physicians, friends and family, and television, and most perceived their diabetes care as an individual as opposed to communal practice. Furthermore, no information on exercise and weight loss was presented with nutrition counseling. Second, treatment was a social, economic, and systemic challenge. Socially, families and friends affected what and how people prepared and consumed food. Financial concerns affected women’s prioritization of the public clinic and dependence on government-funded diabetes medicines. Although women preferred the public system, systemic barriers continued to plague their healthcare experiences. Finally social support influenced how women perceived their diabetes, adhered to diabetes care regimens (such as diets and medication), and felt about their illness experience. In what remains, we discuss three key findings that emerged from our data.

First, women’s social relationships influenced how they learned about, cared for, and treated diabetes. A substantial body of research emphasizes how social and family connectivity can influence diabetes management [26]. Our data reveal positive attributes of social networks, such as how having family or friends with diabetes helped women unburden stress associated with diabetes, as well as negative attributes, including how witnessing family members or friends suffer from diabetes caused them to fear their own future and how stigma forced women to conceal their illness from others. Like women in other social contexts [27], gender roles and social stress influenced women’s diabetes self-care; for example, family members’ reluctance to eat diabetes friendly foods impeded women’s ability to eat foods prescribed by their health care providers. Participants relayed having to cook separately from the family and feeling stressed about dietary changes.

Second, like many low-income populations in LMICs [28], access to medicines remains a pressing concern. Indeed, many women preferred private health insurance schemes that allow patients to receive their medication at home and postpone visits to public facilities where filling prescriptions may require hours of waiting. Although the grievance of public healthcare institutions presenting systemic barriers is not a new one [17, 20], the private model idealized by many women in our study brings to light a low-cost solution to improve diabetes care and reduce cost: home delivery service. The private model of delivering medications by mail to the home was revered by most women who had experienced it, and systemic barriers to accessing medicines was frequently mentioned by those within the public system. In addition, sending a community health worker to patient homes to provide continuing education and social support for maintaining self-care regimens for diabetes related to managing stress, medicines, diets, and exercise could make a big impact. Another low-cost mHealth solution may include utilizing mobile phones with support messaging to remind women about their medication [21].

Finally, our data bring to light the importance of nurses’ and doctors’ messages around diabetes care. Due to the overburdened health system [6], primary care practitioners, including nurses and doctors rarely have enough time with patients to educate comprehensively about diabetes care [18]. Other methods to convey such information should be implemented, such as group counseling sessions to inform patients and quell fears. This strategy would disseminate proper information and utilize limited heath staff more efficiently. Doctors should support mid-level health providers, such as nurse practitioners, to carry out routine NCD care [29]. Increasing the number of nurses available to treat the escalating number of patients with NCDs as well as those with chronic infectious diseases, such as HIV/AIDS, is a step the government must make to improve primary care. However, funding structures must reform to invest in building a stronger health system that can support comprehensive primary care [28]. Indeed, South Africa’s plans for a National Health Insurance may facilitate greater access to private health care facilities and programs, and may address some of the concerns identified for people living with diabetes.

This study is not without limitations. We interviewed a convenience sample of women participating in a cohort study who self-reported diabetes, so these findings may not reflect the experience of people seeking primary care. As with all research of subjective experiences, there is also a possibility of recall bias. Moreover, this is a cross-sectional mixed qualitative and survey study, so we are also unable to say if the perceptions and experiences of the women described by our sample resemble those of people with diabetes only, all Black South Africans residing in Soweto, or other social and economic groups in South Africa. However, our study provides useful insight into navigating diabetes care in South Africa.

## Conclusion

This study makes a contribution to understanding how women experience diabetes care in urban South Africa during a critical period of health systems restructuring. The study indicates that inconsistencies across public and private health systems with regards to diabetes counseling, drug availability, quality of care, and patient wait times influence patient frustration and lack of confidence in the public health care system. Many patients forego a clinical visit in lieu of diabetes self-management to avoid the over-burdened system (which may lead to poorer glucose control), indicating that innovative approaches and community-based care is critical for this population. Such approaches will also address social and financial barriers to good diabetes management. With a stronger national emphasis on the needs of low-income patients with diabetes, health systems can better mobilize human resources, medication, and community education in order to mitigate the growing epidemic.
